# Identifying ADGRG1 as a specific marker for tumor-reactive T cells in acute myeloid leukemia

**DOI:** 10.1186/s40164-024-00560-0

**Published:** 2024-09-06

**Authors:** Yihan Mei, Yu Liu, Wenbing Liu, Manling Chen, Xiaoyu Liu, Shangshang Wang, Junli Mou, Haiyan Xing, Kejing Tang, Zheng Tian, Qing Rao, Min Wang, Runxia Gu, Shaowei Qiu, Jianxiang Wang

**Affiliations:** 1 State Key Laboratory of Experimental Hematology, National Clinical Research Center for Blood Diseases, Institute of Hematology & Blood Diseases Hospital, Chinese Academy of Medical Sciences & Peking Union Medical College (CAMS&PUMC), Tianjin, 300020 China; 2 Tianjin Institutes of Health Science, Tianjin, 301600 China

**Keywords:** AML, Tumor-reactive T cell, ADGRG1

## Abstract

Besides chemotherapy and hematopoietic stem cell transplantation (HSCT), autologous T cells can also serve as a new treatment approach for AML patients. However, the features of tumor-reactive T cells and their distinctive markers still lack full description. To evaluate the characteristics of tumor-reactive T cells, we collected bone marrow (BM) T cells from newly diagnosed AML patients with *RUNX1::RUNX1T1* as examples for paired single-cell RNA sequencing and single-cell V(D)J sequencing. Based on the STARTRAC-like algorithm, we defined bystander T cells and tumor-reactive T cells. Compared with bystander T cells, tumor-reactive T cells presented as senescent-like cytotoxic terminally differentiated T cells (Temra) with upregulated NK-related markers. Additionally, we found *ADGRG1* could serve as the specific marker of CD8^+^ T tumor-reactive T cell and validated it through the Runx1^Runx1t1/+^; Mx1-Cre mouse model. In chimeric antigen receptor (CAR)-T and target cell system, ADGRG1 was selectively upregulated upon antigen-TCR encounter. Moreover, ADGRG1^+^CD8^+^ T cells released a higher level of IFN-γ and showed higher cell-killing ability when exposed to matched AML blasts. Together, our findings depict the single-cell profile of tumor-reactive T cells in AML BM and propose that ADGRG1 can act as an indicator of T cell tumor reactivity in AML, which may be further harnessed for adoptive cell therapy and tumor-reactive TCR enrichment.

**Supplementary Information:**

The online version contains supplementary material available at 10.1186/s40164-024-00560-0.

## Key points

1. CD8^+^ tumor-reactive T cells present in AML BM and are characterized as senescent-like cytotoxic Temra with upregulated NK-related markers.

2. ADGRG1 can serve as a specific marker of CD8^+^ tumor-reactive T cells in AML BM.

**Supplementary Information:**

The online version contains supplementary material available at 10.1186/s40164-024-00560-0.

## Introduction

Immunotherapy, especially T-cell based therapy, has shown promising clinical efficacy in the treatment of solid tumors [1–3]. These advances are driven by the growing understanding of tumor-reactive T cells in solid tumors [4, 5]. They mainly exhibited the exhausted profile with upregulated inhibitory immune checkpoint receptors like PD-1 [6]. Among the exhausted T cell pool, the precursor-exhausted CD8^+^ T cells respond to the immune checkpoint blockade (ICB) therapy and closely correlate with its duration and efficacy [7, 8].

However, immunotherapy like ICB and T cell receptor-engineered T cell (TCR-T) produce unsatisfactory clinical improvement in acute myeloid leukemia (AML). For example, early phase 1 trials based on anti-PD-1 monoclonal antibody as monotherapy show lack of efficacy [9, 10]. Even combined nivolumab with azacytidine, the remission rate in relapse/refractory AML is still limited [11].

The reason for such divergent therapeutic outcomes may stem from the unique profile of T cells in AML. Studies in other hematological malignancies indicate the distinctive T cell landscape, such as T cells in multiple myeloma display the feature of senescence [12] and T cells in Waldenstrom macroglobulinemia exhibit anergic properties [13]. But the functional state of T cells in AML, especially the CD8^+^ tumor-reactive T cells, remains elusive. Nevertheless, some researchers have found tumor-reactive TCR sequences in AML with genomic abnormalities, such as core-binding factor AML [14]. To this end, it is necessary to illustrate tumor-reactive T cells in AML, thus aiding AML immunotherapeutic treatment.

In this study, we collect 5 BM samples from newly diagnosed AML patients with *RUNX1::RUNX1T1*, one of the most common genetic abnormalities in core-binding factor AML [15, 16], as examples for paired scRNA-seq and scV(D)J-seq. We trace the differentiation trajectory of tumor-reactive T cells and reveal that the AML tumor-reactive T cell shows a non-exhausted senescent-like cytotoxic T cell profile with upregulated NK-related markers. We identify *ADGRG1* as the specific marker of tumor antigen-experienced CD8^+^ T cell and validate it through the conditional mouse model. The in vitro experiments such as the interferon-γ (IFN-γ) release assays and the cell-killing assay using AML samples also confirm the tumor-reactive property of ADGRG1^+^CD8^+^ T cells. Thus, ADGRG1 may be further harnessed for adoptive cell therapy and tumor-reactive TCR enrichment in AML.

## Methods

### Human and mouse samples

The AML patients’ BM samples involved in this study were obtained from the Institute of Hematology & Blood Diseases Hospital, Chinese Academy of Medical Science, and Peking Union Medical College. The available clinical characteristics are summarized in Supplementary Table S1. Written informed consent was provided by all patients. All experimental procedures involving humans were reviewed and approved by the ethics committee. The Runx1^Runx1t1/+^; Mx1-Cre mouse model was constructed and all mouse-related experimental procedures underwent review and approval by the ethics committee. The detailed information of mouse model generation is shown in Supplementary methods.

### Single-cell library preparation, sequencing, and data analysis

The prepared human and mouse samples were sorted for the following experiments (Supplementary methods). Single-cell RNA-seq libraries were prepared using the 10× Chromium Single-cell 5’ and VDJ library construction (10× Genomics), following the manufacturer’s instructions. Purified libraries were subject to NovaSeq 6000 sequencer (Illumina) for paired-end (150 bp) sequencing. The gene count matrix was created using the Cell Ranger toolkit (version 6.1.2, https://www.10xgenomics.com/support/software/cell-ranger/latest). Subsequent analysis steps were primarily conducted using Scanpy (version 1.9.1) [17] and Scirpy (version 0.11.0) [18], including quality control, normalization, logarithmical transformation, TCR clonotype definition, dimension reduction, unsupervised clustering, and calculation of differentially expressed genes (DEGs). The pseudotime was calculated by Palantir (version 1.3.2) [19]. The transcriptional regulon analysis was performed by pySCENIC (version 0.12.1) [20]. The pTRT identification process and other detailed analysis workflow are provided in the Supplementary Methods.

### CAR-T cell coculture assay

The detailed protocol of human CD3^+^ T cell isolation, activation, and CAR-T generation has been described previously [21]. In brief, T cells from healthy donor’s peripheral blood were isolated and activated with Dynabeads™ Human T-Activator CD3/CD28 (Gibco, Cat# 11161D) and recombinant human interleukin (rIL)-2 (R&D, Cat# 202-IL). After stimulation for 24 h, T cells were transduced with lentiviral supernatants and replaced with fresh culture medium after 48 h. On day 7 after stimulation, T cells were cocultured with cell lines (Molm13 or K562) at an effector: target (E: T) ratio of 1:1 for 48 h. Then cell markers were detected by flow cytometry. Anti-human antibodies used for surface markers are APC anti-CD3 (Biolegend, Cat# 317317), PerCP anti-CD8 (Biolegend, Cat# 300921), and PE/Cyanine7 anti-ADGRG1 (Biolegend, Cat# 358205).

### IFN-γ releasing assay

The ADGRG1^+^CD8^+^ T cells and ADGRG1^−^CD8^+^ T cells and blast cells were sorted from newly diagnosed AML bone marrow samples by BD FACSAria™ III sorter (BD Biosciences). 2 × 10^3^ T cells were then cocultured with matched blasts in a 96-well plate with rIL-2 at an E: T ratio of 1:1 for 48 h in 200 µl medium. After 400G centrifugation for 5 min, the supernatants were collected and the concentration of IFN-γ was quantified by the capture of microspheres encapsulated with cytokine-specific antibodies (Cellgene Biotech, Cat# P420487) using flow cytometry. This quantification was based on the fluorescence intensity of the complexes, expressed in pg/ml.

### Cell killing assay

The mononuclear cells were isolated by Ficoll-Paque PREMIUM density gradient medium (Cytiva, Cat# 17544202). For further sorting, isolated cells were stained by APC/Cy7 anti-human CD45 antibody (Biolegend, Cat# 368515), APC anti-human CD34 antibody (Biolegend, Cat# 378605), PerCP anti-CD8 antibody (Biolegend, Cat# 300921), PE/Cyanine7 anti-ADGRG1 antibody (Biolegend, Cat# 358205), and DAPI (Sigma-Aldrich, Cat# D9542). ADGRG1^+^CD8^+^ T cells, ADGRG1^−^CD8^+^ T cells, and blast cells were sorted from each patient. Then ADGRG1^+/−^CD8^+^ T cells (∼ 2 × 10^4^/well in a 96-well plate) were cocultured with matched blasts with an E: T ratio of 1:1 for 24 h. After 24 h coculture, cells were stained by APC/CY7 anti-human CD3 antibody (Biolegend, Cat# 300317) and PE anti-human CD123 antibody (Biolegend, Cat# 983706). The CD3^−^CD123^+^ cells were residual blasts and the absolute count was calculated by CountBright™ absolute counting beads (Thermo Fisher, Cat# C36950). The cell killing ability was measured by $\frac{Absolute\:count\:of\:residual\:blast\:cells\:at\:24h}{Absolute\:count\:of\:blast\:cells\:at\:0h}$.

### Quantification and statistical analysis

Statistical analyses were performed using GraphPad Prism (version 9.4.0) and Python (version 3.9.9). Graphs were mainly generated using GraphPad Prism and Matplotlib (version 3.5.1) package. All error bars were reported as mean ± SEM. The level of significance was indicated as p-value: ns > 0.05, * < 0.05, ** < 0.01, *** < 0.001, **** < 0.0001.

## Results

### Single-cell characterization of T cells in newly diagnosed AML patients with *RUNX1::RUNX1T1*

**Fig. 1:**
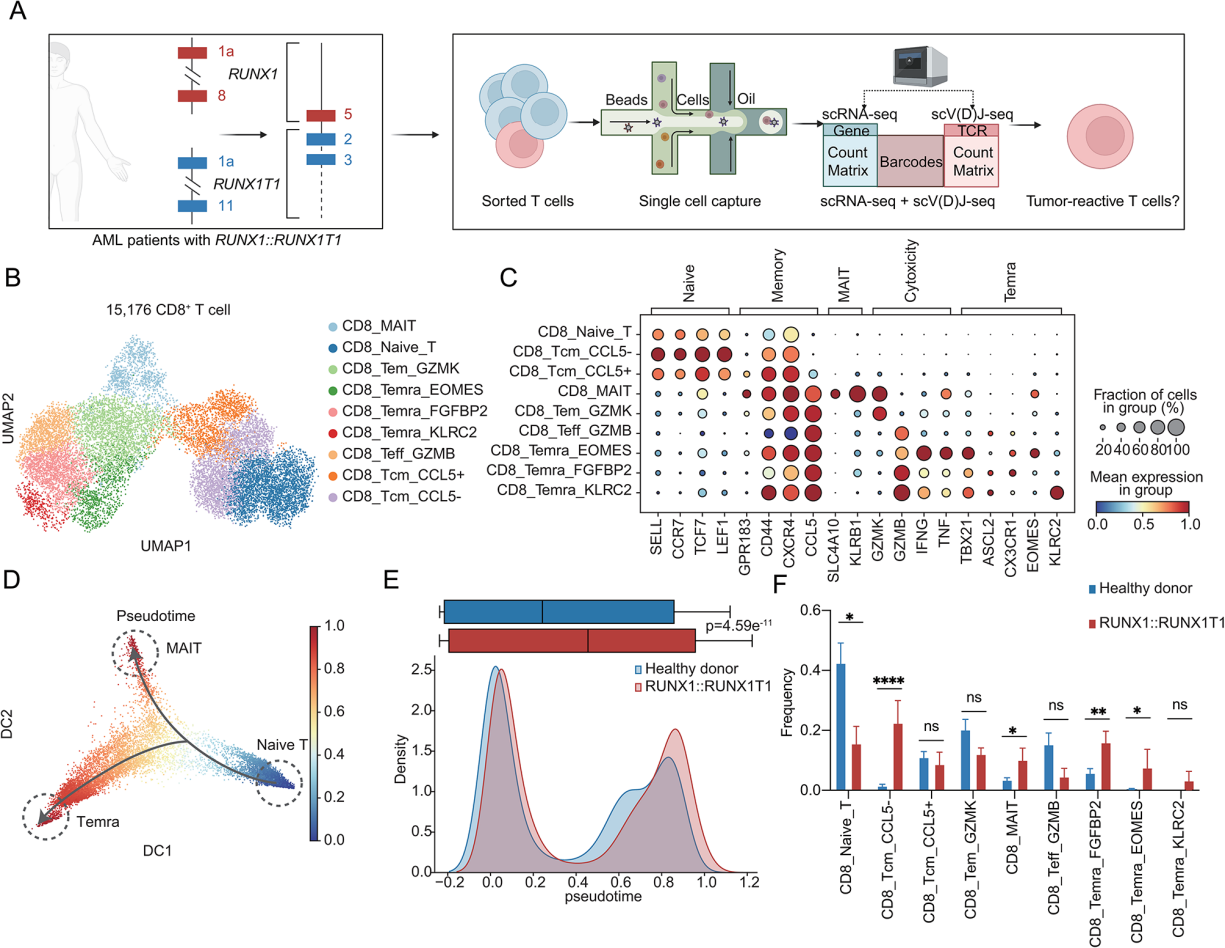
The single cell profile of T cells from newly diagnosed AML patients’ BM with *RUNX1::RUNX1T1*. **A** Schematic overview of the study. BM samples were collected from 5 AML patients with *RUNX1::RUNX1T1*. The CD3^+^ T cells were sorted for scRNA-seq and scV(D)J-seq. This figure is edited by BioRender.com. **B** UMAP visualization of identified 9 CD8^+^ T cell clusters. The subgroups corresponding to each color in the UMAP plot are indicated in the right-side legend. **C** Dot plot showing the relative expression of marker genes across different CD8^+^ T cells. Bubble size is proportional to the percentage of cells expressing a gene and color intensity is proportional to average scaled gene expression. **D** The diffusion map of CD8^+^ T cells revealing a continuum of cellular states. The color shows the pseudotime performed by Palantir [19]. **E** Comparison of the pseudotime (calculated with Palantir) between CD8^+^ T cells in RUNX1::RUNX1T1 (red) and HD (blue) group showed by boxplot (top) and density plot (bottom). The significance was calculated by the Wilcoxon rank-sum test. **F** Boxplot showing the proportion of CD8^+^ T cell in the RUNX1::RUNX1T1 group (red) and the HD (blue) group. Student’s t-test was used to measure the differences between the two groups. p-value: no significance (ns) > 0.05, * ≤ 0.05; ** ≤ 0.01; ***≤ 0.001, **** ≤ 0.0001

To explore the heterogeneity of T cell compartment and the characteristics of tumor-reactive T cells, we collected BM T cells from 5 newly diagnosed AML patients with *RUNX1::RUNX1T1* and performed scRNA-seq and scV(D)J-seq (Fig. 1A). AML patients’ characteristics are described in Supplementary Table S1. To identify potentially tumor-reactive T cells (pTRTs) and help cluster annotation of the BM T cell landscape, we integrated a T cell reference from 20 healthy donors [22] (HDs) with AML patients’ data. In total, 57,186 T cells (25,868 cells from AML patients and 31,318 cells from HDs) were integrated after quality control, doublet removal, and batch correction (Supplementary Figure S1A). In addition, to evaluate T cell activities in AML patients, we performed scV(D)J-seq and identified paired productive ɑ and β chain in 22,214 T cells.

Based on classical gene markers, we defined 15,176 CD8^+^ T cells and annotated unsupervised clusters (Fig. 1B-C, Supplementary Figure S1B). Overall, the CD8^+^ T cells could be classified into various cell types, including CD8^+^ naïve T cells (Tn, *CCR7*), CD8^+^ central memory T cells (Tcm, naïve markers and *CD44*), CD8^+^ effector memory T cells (Tem, *GZMK*) CD8^+^ effector T cells (Teff, *GZMB*), CD8^+^ mucosal-associated invariant T cells (MAIT, *SLC4A10*, *TRAV1-2*, Supplementary Figure S1C), and CD8^+^ terminally differentiated effector T cells (Temra, *TBX21*). Through trajectory inference, we discovered that T cells differentiated into two pathways: from naive T cells to MAIT or Temra (Fig. 1D, Supplementary Figure S1D). Compared to healthy donors, T cells in AML BM were significantly more advanced in pseudotime (Fig. 1E, Supplementary Figure S1E). From the distribution of cell clusters, we found that the total percentage of Tn in the RUNX1::RUNX1T1 group was much lower (15.56% vs. 42.45%, *p* = 0.04), while the percentage of Temra increased (CD8_Temra_FGFBP2: 15.95% vs. 5.72%, *p* = 0.007; CD8_Temra_EOMES: 7.52% vs. 0.42%, *p* = 0.04).

In addition, we also defined the 5,555 CD4^+^ T cells based on classical gene markers (Supplementary Figure S1F-I). Similarly, we could also observe a decrease in Tn (16.45% vs. 73.44%, *p* < 0.0001) and an increase in more differentiated clusters of RUNX1::RUNX1T1 group (CD4_Tcm: 42.65% vs. 8.30%, *p* = 0.0007; CD4_Tem_LMNA: 24.43% vs. 5.97%, *p* = 0.004).

These data suggested that, in the BM of AML patients with *RUNX1::RUNX1T1*, the immune reserve reduced and differentiated T cells increased, indicating immune mobilization.

### The pTRTs exhibited heterogeneity in AML patients with *RUNX1::RUNX1T1*

**Fig. 2:**
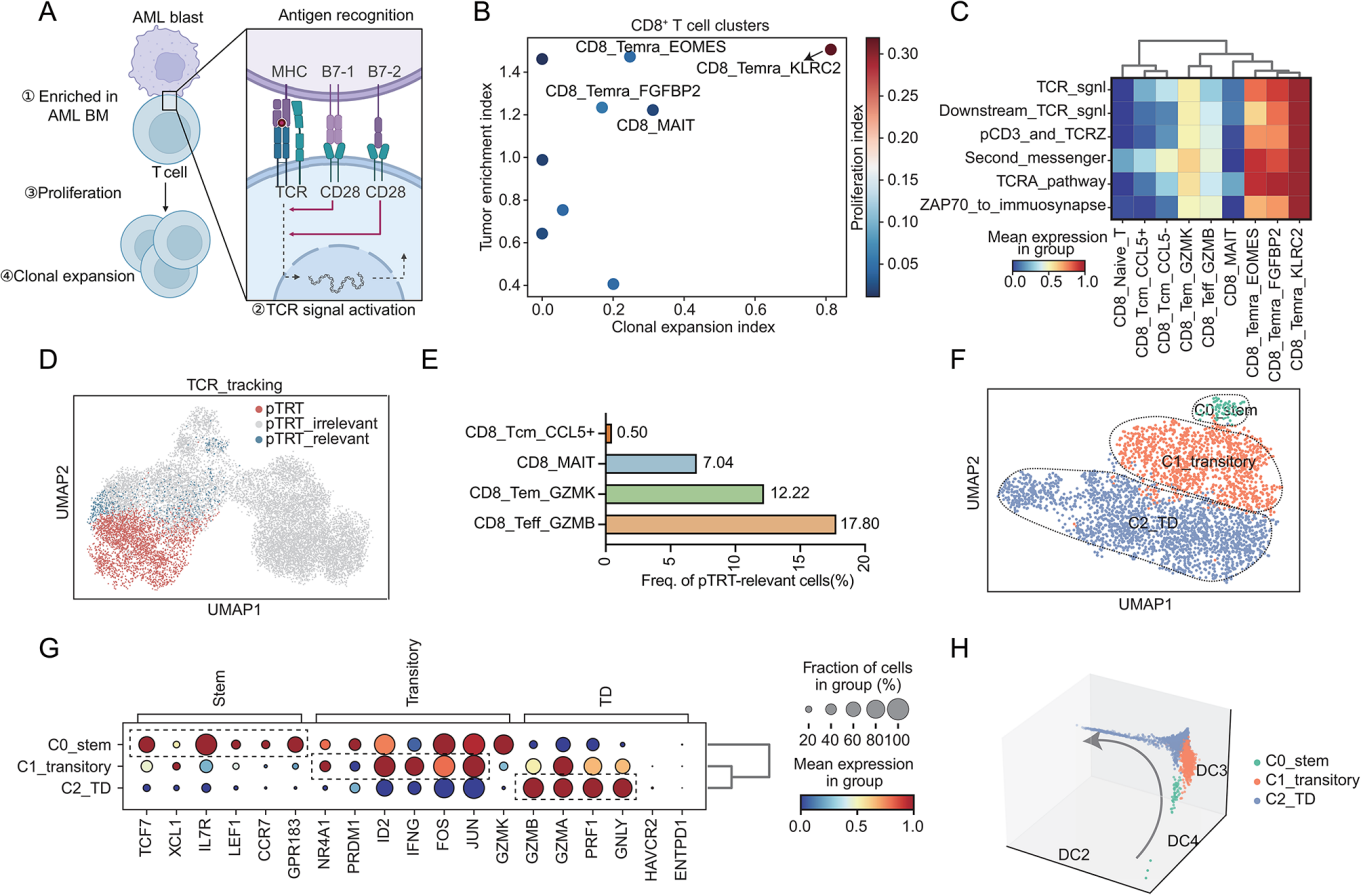
The identification and characteristics of tumor-reactive T cells in AML. **A** The schema plot for identification of pTRTs. This figure is edited by BioRender.com. **B** The scatter plot of the tumor enrichment index and clonal expansion index in each CD8^+^ T cell cluster. The proliferation index is shown in gradient color and the color bar is annotated on the right margin. **C** Heatmap showing the TCR downstream signal activation level of different clusters. TCR-signaling-related genesets were obtained from https://www.gsea-msigdb.org/gsea/msigdb/. TCR_sgnl: REACTOME_TCR_SIGNALING, Downstream_TCR_sgnl: REACTOME_DOWNSTREAM_ TCR_SIGNALING, pCD3_and_TCRZ: REACTOME_PHOSPHORYLATION_OF_CD3_AND_TCR_ZETA_CHAINS, Second_messenger: REACTOME_GENERATION_OF_SECOND_MESSENGER_MOLECULES, TCRa_pathway: BIOCARTA_TCRA_PATHWAY, ZAP70_to_immuosynapse: REACTOME_TRANSLOCATION_OF_ZAP_70_TO_IMMUNOLOGICAL_SYNAPSE. **D** UMAP visualization of clusters annotated by TCR repertoire. Red represents pTRT, blue represents pTRT-relavant cells and the rest of the cells are annotated as pTRT-irrelevant cells in grey. **E** Bar plot of pTRT-relevant cell frequency in each cluster. **F** The UMAP plot of tumor-reactive T cells. C0_stem: stem-like cluster, C1_transitory: transitory cluster, C2_TD: terminally differentiated cluster. **G** Dot plot showing the relative expression of marker genes across different tumor-reactive T cell clusters. Bubble size is proportional to the percentage of cells expressing a gene and color intensity is proportional to average scaled gene expression. **H** The diffusion map of tumor-reactive T cells. The color annotations are on the right margin. The differential trajectory is indicated by the grey arrow

Since AML patients exhibited abnormal distribution of T cell proportions in BM, it suggested that T cells might be activated in the presence of tumor antigen stimulation, indicating the presence of pTRTs. As pTRTs, they should be selectively enriched in the AML microenvironment, with TCR capable of recognizing AML tumor antigens and activating downstream signaling pathways, leading to T cell proliferation and clonal expansion (Fig. 2A). Therefore, to distinguish pTRTs from bystander T cells, we used four criteria mainly applied from STARTRAC [23], namely tumor enrichment index (Supplementary Figure S2A), clonal expansion index (Supplementary Figure S2B), proliferation index (Supplementary Figure S2C), and TCR signaling pathway activation [5] (Fig. 2B-C). Based on the four characteristics, we identified three Temra clusters (CD8_Terma_FGFBP2, CD8_Temra_EOMES, and CD8_Temra_KLRC2) as pTRTs and validated them by published gene sets [24] (Supplementary Figure S2D).

To explore the origin of pTRTs, we conducted TCR-based tracing. We defined a group of cells that shared TCR with pTRTs as pTRT-relevant cells (Fig. 2D). The main components of pTRT-relevant cells belong to CD8_Teff_GZMB (17.80%) and CD8_Tem_GZMK (12.22%), which are in line with pan-cancer analysis results [5] (Fig. 2E).

Next, we performed unsupervised clustering on pTRTs and pTRT-relevant cells and defined three clusters based on different gene signatures [25] (Fig. 2F). The stem-like C0 cluster exhibited a relatively high level of naïve markers like *TCF7* and *IL7R* (Fig. 2G) which was mainly composed by CD8_Tem_GZMK (Supplementary Figure S2E-F). The transitory C1 cluster was characterized by acute-TCR-engagement-related genes (*NR4A1*, *FOS*, and *JUN*) [26] and effector makers. Compared to C1, C2 exhibited reduced expression of transitory genes and high expression of effector markers and was thus defined as the terminally differentiated (TD) cluster (Fig. 2G). Additionally, *GZMK* was highly expressed in C0, while *GZMB* was highly expressed in C1/C2, suggesting that differential expression of granule enzymes could reflect the developmental state of cells and serve as markers.

Therefore, our results indicated the existence of tumor-reactive T cells (pTRTs and pTRT-relevant cells) in the BM of AML patients carrying the *RUNX1::RUNX1T1* which showed a continuous spectrum of differentiation from the stem-like cluster to the terminally differentiated cluster (Fig. 2H, Supplementary Figure S2G).

### The tumor-reactive T cells showed distinct non-exhausted cytotoxic transcriptional features in AML

**Fig. 3:**
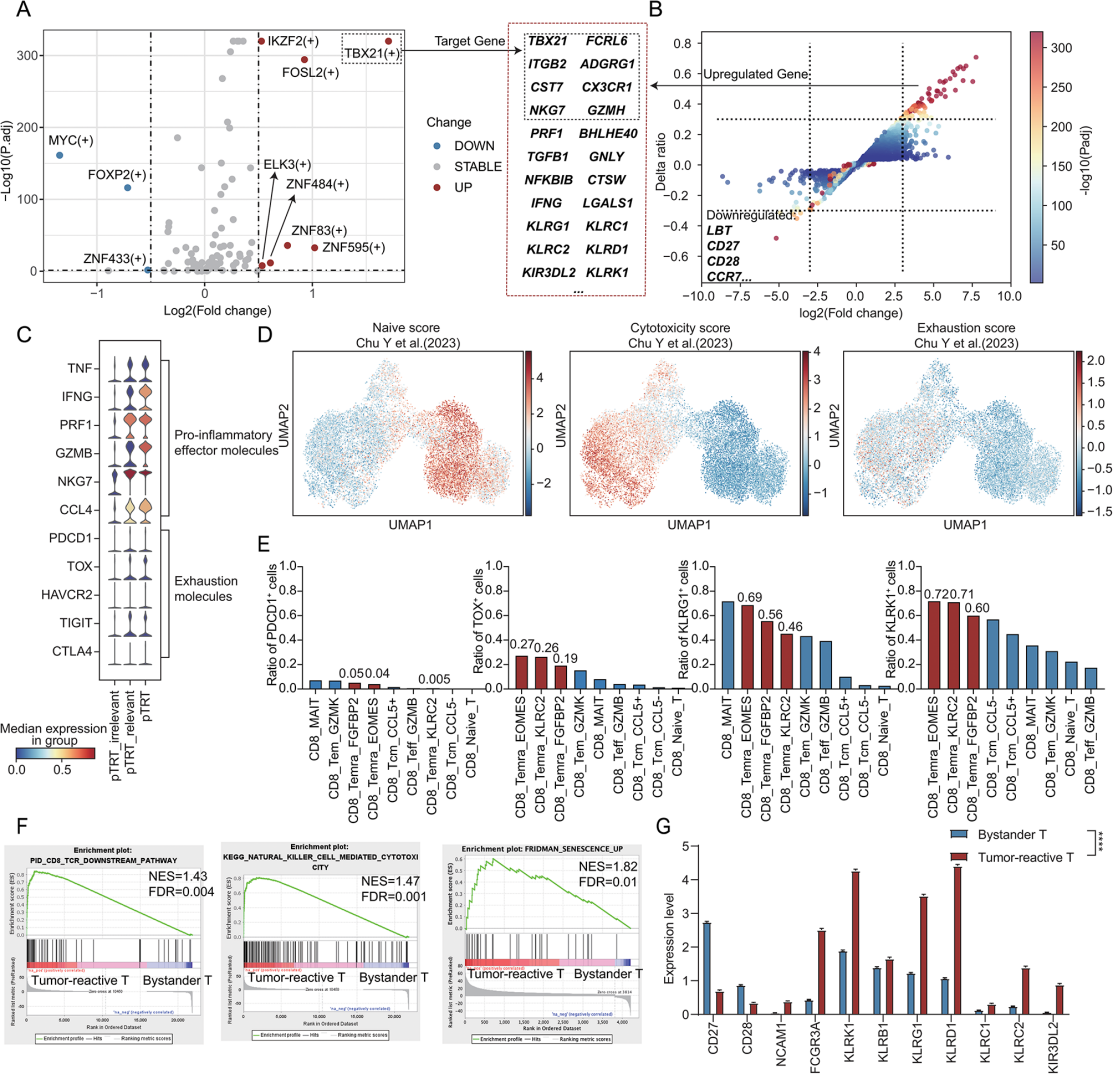
Comparing the characteristics between tumor-reactive T cells and bystander T cells. **A** Volcano plot showing the specifically activated regulons in the tumor-reactive and bystander T cells. The upregulated target genes are shown in the black dashed box. The vertical dashed line represents a log2 (fold change) value of 0.5, and the horizontal dashed line represents an adjusted p-value of 0.05. **B** Scatter plot showing the differentially expressed genes in tumor-reactive and bystander T cells. The x-axis represents the log2 (fold change) value with a threshold value of 3, and the y-axis represents the difference in expression ratios of genes between tumor-reactive T cells and bystander T cells (threshold value: 0.3), with the color of the points indicating the -log10 (p-value). The upregulated genes are partly shown in the red dashed box. **C** The stacked violin plot showing the pro-inflammatory effector molecules and exhaustion-related molecules expression in tumor-reactive T cells and bystander T cells. **D** The UMAP plot illustrating the naïve/cytotoxicity/exhaustion score in CD8^+^ T cells. The color bar is shown on the right margin. **E** Proportions of *TOX*/*PDCD1/KLRG/KLRK*-positive cells in each T cell cluster. The pTRT-related Temra clusters are drawn in red, while the rest of the clusters are in blue. **F** Representative pathways enriched in the identified genes as determined by gene set enrichment analysis (GSEA) [57]. The normalized enrichment score (NES) and the false discovery rate (FDR) are annotated in the top right corner. **G** The bar plot showing the senescence-like/NK-related gene expression between bystander T cells (blue) and tumor-reactive T cells (red). The Wilcoxon rank sum test was performed for calculation. The p-values for each gene are all less than 0.0001

To address the molecular characteristics of tumor-reactive T cells in AML with *RUNX1::RUNX1T1*, we compared the transcriptional regulon (Fig. 3A) and gene expression profile (Fig. 3B, Supplementary Table S2) between tumor-reactive T cells and bystander T cells. The transcriptional programs regulated by *TBX21*, *ZNF595*, *FOSL2*, *ZNF83*, *ZNF484*, *IKZF2*, and *ELK3* were found to be significantly activated in tumor-reactive T cells. The *TBX21* regulon, whose target genes included *ADGRG1*, *CST7*, *CX3CR1*, and so on, was identified as a Temra-specific regulon in CD8^+^ T cells [27]. The *FOSL2* was an AP1 family member, functioning downstream of TCR signaling and driving CD8^+^ effector differentiation [28]. Consistent with the regulon profile, tumor-reactive T cells exhibited higher expression of pro-inflammatory effector molecules such as *IFNG*, *TNF*, and *PRF1* (Fig. 3C) and lower expression of genes like *LBT*, *CD27*, and *CD28*.

Interestingly, tumor-reactive T cells demonstrated high cytotoxicity scores and low exhaustion scores (Fig. 3D). The expression levels (Fig. 3C) and proportions (Fig. 3E) of exhaustion-related markers like *PDCD1* and *TOX* were also low. This suggested that tumor-reactive T cells in AML displayed a unique non-exhausted but cytotoxic profile compared to solid tumors.

Next, we performed functional enrichment analysis on tumor-reactive T cells and found enrichment of the TCR downstream pathway validating the feature of tumor reactivity. Notably, the NK-mediated cytotoxicity pathway was enriched (Fig. 3F) and NK-related makers like *KLRG1* and *KLRK1* were significantly upregulated (Fig. 3B, Fig. 3E, Fig. 3G). These tumor-reactive cells did not express invariant Vα24-Jα18 chain and Vβ11 chain (Supplementary Figure S3), which excluded the possibility of invariant NKT (iNKT) [29].

Previous studies have indicated that Temra cells lacking *CD27*/C*D28* expression and expressing NK-related markers such as *KLRG1* exhibit a senescent-like but functionally active profile after antigen stimulation [30–34] and our results also confirmed such characteristics (Fig. 3F-G). Therefore, the tumor-reactive T cells in AML exhibited a specific non-exhausted cytotoxic Temra profile with upregulated NK-related molecules.

### The *ADGRG1* was highly and almost specifically expressed in tumor-reactive T cells

**Fig. 4:**
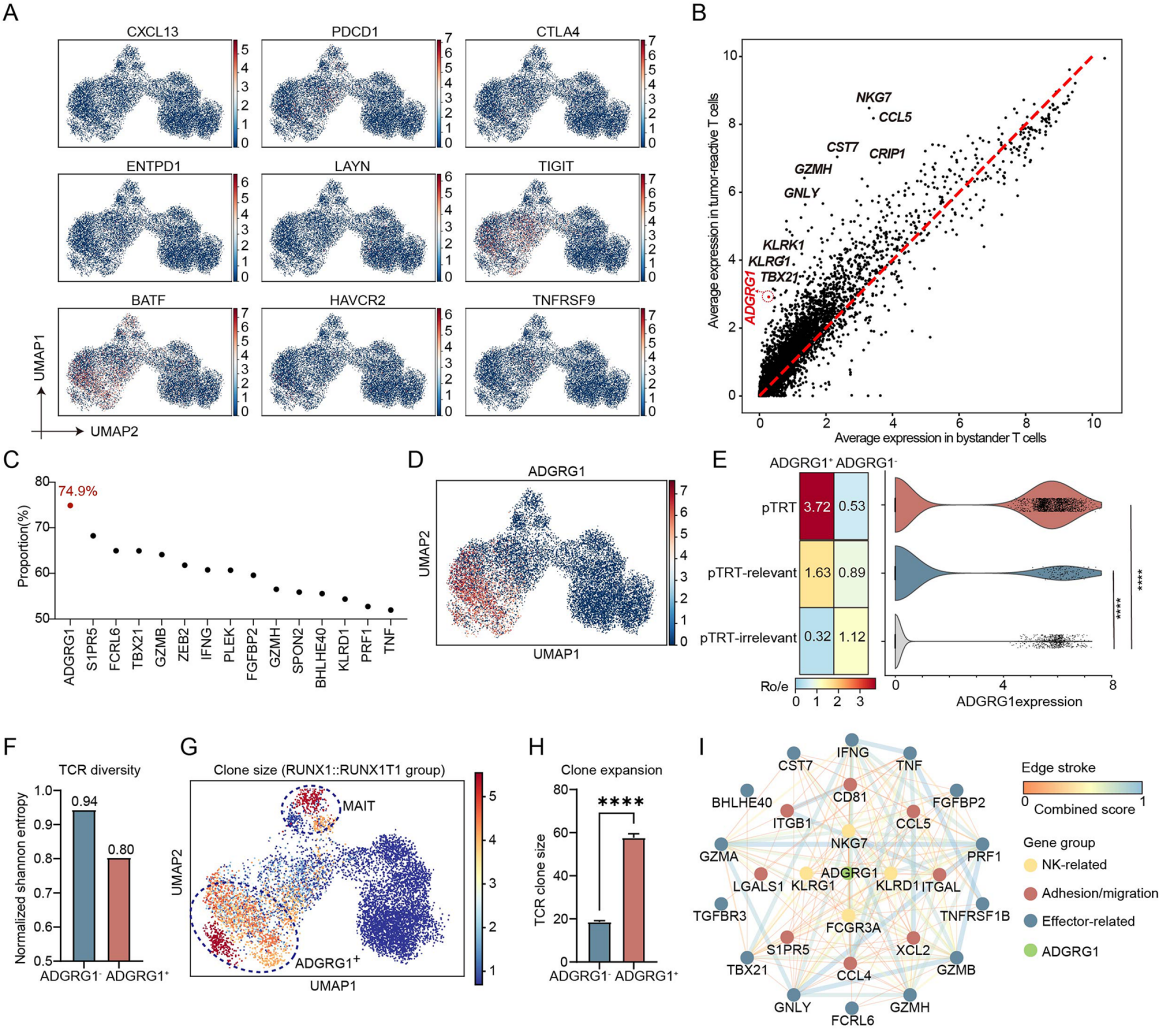
The tumor-reactive T cells expressed a high level of *ADGRG1*. **A** The feature plots showing the expression levels of tumor-reactive T cells’ marker genes in solid tumors. The color bars for each graph are placed on their respective right margin. **B** Scatter plot showing the average gene expression of tumor-reactive T cells versus bystander T cells. The horizontal axis represents the average expression level of genes in bystander T cells, while the vertical axis represents the average expression level in tumor-reactive T cells. The 45-degree red dashed line indicates where the average expression levels are equal in both cell types. Some gene names that are highly expressed in tumor-reactive T cells are labeled near their corresponding points. **C** The frequency of pTRTs in certain gene expressed cells. The *ADGRG1* gene is shown in red dot. **D** The UMAP plot visualizing the *ADGRG1* expression in CD8^+^ T cells. **E** The distribution pattern of ADGRG1^+/−^CD8^+^ T cells. (Left) matrix plot showing distribution prevalence estimated by Ro/e. (Right) violin plot showing the *ADGRG1* expression among pTRT, pTRT-relevant, and pTRT-irrelevant groups. **F** Bar plot showing the normalized Shannon entropy of ADGRG1^−^ and ADGRG1^+^ cells. **G** UMAP plot of the log-transformed clone size value in the RUNX1::RUNX1T1 group. The color bar is shown on the right side. **H** Bar plot showing the clone size of ADGRG1^−^ and ADGRG1^+^ cells. **I** The functional module involved by *ADGRG1* based on STRING [58]. The edge stroke color is scaled by combined score which was computed by combining the probabilities from the different evidence channels and corrected for the probability of randomly observing an interaction [59]. The gene group annotation is shown on the right margin. In Fig. 4E and H, the Wilcoxon rank-sum test was applied to calculate the p-value (**** ≤ 0.0001)

In solid tumors, the tumor-reactive T cells are characterized by high expression levels of *CXCL13* or exhaustion signatures [35]. However, tumor-reactive T cells in AML did not exhibit significant expression of *CXCL13* or classical exhaustion molecules (Fig. 4A). To explore the distinctive maker of tumor-reactive T cells in AML, we examined the average gene expression pattern of tumor-reactive T cells and bystander T cells (Fig. 4B). Genes such as *ADGRG1*, *TBX21*, *S1PR5*, *FCRL6*, and *FCGR3A* showed relatively selective expression in tumor-reactive T cells (the average expression in bystander T cells < 0.5, Supplementary Table S3). For the top 100 genes based on the delta value (Supplementary Methods), we calculated the proportion of tumor-reactive T cells among the cells expressing those genes (Fig. 4C, Supplementary Table S4). Particularly, we found that *ADGRG1* had the highest proportion (74.90%) and was almost specifically expressed in tumor-reactive T cells (Fig. 4D-E). Additionally, ADGRG1^+^ cells exhibit reduced TCR clone diversity (Fig. 4F) and a significant increase in TCR clone size (Fig. 4G-H), indicating a state of clonal expansion.

ADGRG1, also known as GPR56, is a member of the adhesion G protein-coupled receptor (GPCR) family. *ADGRG1* is expressed in NK cells, gdT cells, and terminal effector T cells in healthy human BM (Supplementary Figure S4A-B) [22, 36]. In mouse, Adgrg1 is expressed in hematopoietic stem/progenitor cells (Supplementary Figure S4C-D) [37, 38]. Notably, ADGRG1 has also been found to serve as a marker for leukemia stem cells [39]. Recent studies have revealed that ADGRG1^+^CD8^+^ T cells, following allogeneic HSCT, function as allo-reactive cytotoxic T cells that are capable of recognizing the patients’ original AML blasts [40]. By performing network analysis, we demonstrated that the upregulated genes in the ADGRG1^+^ group like *IFNG*, *S1PR5*, and *CD81* were involved in a functional module with *ADGRG1* (Fig. 4I), indicating the relation between *ADGRG1* and T cell effector function.

Thus, we observed an elevated expression of *ADGRG1* specifically in T cells of AML, particularly in tumor-reactive T cells, suggesting its potential utility as a marker for tumor-reactive T cells.

### The *Adgrg1* was specifically expressed in tumor-reactive T cells in the Runx1::Runx1t1 mouse model

**Fig. 5:**
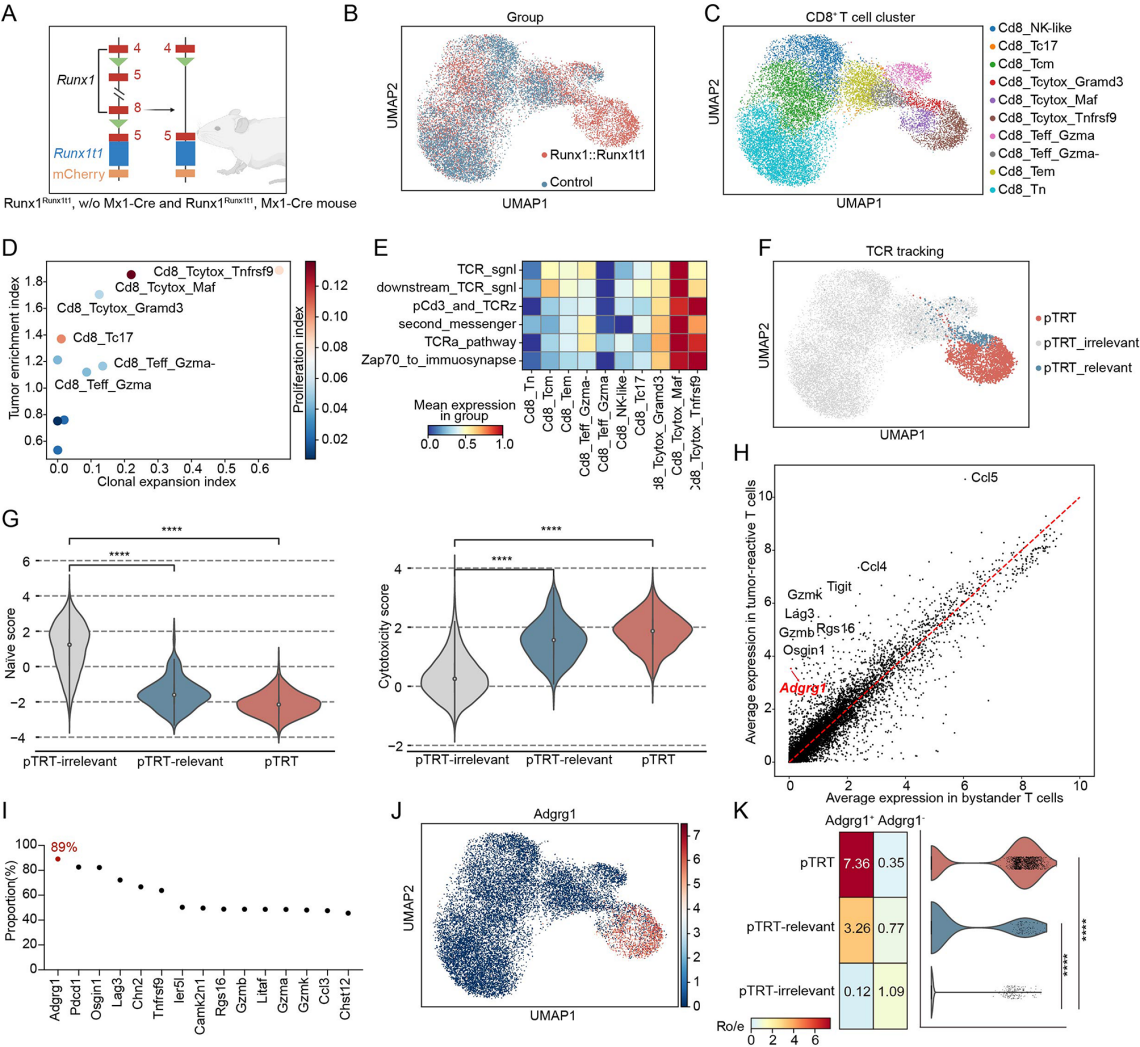
The T cell profile in the BM of Runx1^Runx1t1/+^; Mx1-Cre mouse. **A** Overview of Runx1^Runx1t1/+^; Mx1-Cre mouse model construction. The conditional inducible knock-in C57BL/6 murine model was established with an inducible Cre recombinase (Mx1-Cre) and heterozygous conditional knock-in of *Runx1::Run1t1* in the hematopoietic system (the Runx1::Runx1t1 group), in which the mCherry^+^ cell indicated the *Runx1::Runx1t1* fusion gene expression. The mouse with heterozygous conditional knock-in of *Runx1::Run1t1* and without Mx1-Cre was categorized as the control group which is mCherry^−^. This figure is edited by BioRender.com. **B** UMAP visualization of mouse BM Cd8^+^ T cells. Red represents the Runx1::Runx1t1 group and blue represents the control group. **C** UMAP plot showing the identified 10 Cd8^+^ T cell clusters. The subgroups corresponding to each color in the UMAP plot are indicated in the right-side legend. **D** The scatter plot of the tumor enrichment index and clonal expansion index in each mouse Cd8^+^ T cell cluster. The proliferation index is shown in gradient color and the color bar is annotated on the right margin. **E** Heatmap showing the TCR downstream signal activation level of different mouse Cd8^+^ clusters. The abbreviation of TCR signaling pathways is the same as that in Fig. 2C. **F** UMAP visualization of clusters annotated by TCR repertoire in mouse Cd8^+^ T cells. Red represents pTRT, blue represents pTRT-relevant cells and the rest of the cells are annotated as pTRT-irrelevant cells in grey. **G** The violin plot showing the naïve score (left) and the cytotoxicity score (right) among pTRT, pTRT-relevant, and pTRT-irrelevant groups. **H** Scatter plot showing the average gene expression of tumor-reactive T cells versus bystander T cells in mouse Cd8^+^ T cells. **I** The frequency of pTRTs in certain gene expressed cells. The *Adgrg1* gene is shown in red dot. **J**, The UMAP plot showing the *Adgrg1* expression in mouse Cd8^+^ T cells. **K** The distribution pattern of Adgrg1^+/−^Cd8^+^ T cells. (Left) matrix plot showing distribution prevalence estimated by Ro/e. (Right) violin plot showing the *Adgrg1* expression among pTRT, pTRT-relevant, and pTRT-irrelevant groups. In Fig. 5G and K, the Wilcoxon rank-sum test was applied to calculate the p-value between pTRT/pTRT-relevant group with pTRT-irrelevant group: (**** ≤ 0.0001)

Due to the complexity of patient genetic backgrounds, we established the Runx1^Runx1t1/+^; Mx1-Cre mouse model by conditionally knocking in the *Runx1::Runx1t1* gene (Supplementary methods) with a cleaner and more controlled background to further validate the previous results (Fig. 5A). We sorted mCherry^−^ T cells indicating the absence of *Runx1::Runx1t1* fusion gene expression from Runx1^Runx1t1/+^; Mx1-Cre mouse (the Runx1::Runxt1 group) and Runx1^Runx1t1/+^; w/o Mx1-Cre mouse (the control group), respectively. The freshly sorted cells were profiled by scRNA-seq and scV(D)J-seq from 10× Genomics. We obtained a total of 43,450 high-quality cells after quality control (21,410 from the control group and 22,040 from the Runx1::Runxt1 group) (Fig. 5B). Besides, to examine the T cell patterns, we performed scV(D)J-seq and identified paired productive α and β chains in 35,379 T cells.

Overall, unsupervised clustering identified 10 clusters of Cd8^+^ T cells (Fig. 5C) and 15 Cd4^+^ T cells (Supplementary Figure S5D), each with unique signature genes (Supplementary Figure S5A, Supplementary Figure S5E-F). Cd4^+^ T cells and Cd8^+^ T cells both exhibited a reduction in immune reserve cells like Tn and an increase in terminally differentiated T cells like cytotoxic T cells (Tcytox, Supplementary Figure S5B, Supplementary Figure S5G).

Following the steps described in patients’ data analysis, we identified pTRTs in the BM of Runx1^Runx1t1/+^; Mx1-Cre mouse, including the Cd8_Tcytox_Maf and Cd8_Tcytox_Tnfrsf9 cluster, which were specifically TCR activated and clonal expanded in the Runx1::Runx1t1 group (Fig. 5D-F). Tumor-reactive T cells in the Runx1^Runx1t1/+^; Mx1-Cre mouse model also exhibited reduced naïve score, high cytotoxic score, and high senescence score (Fig. 5G, Supplementary Figure S5C).

To explore the gene specifically expressed in tumor-reactive T cells, we compared the gene expression level between tumor-reactive T cells and bystander T cells (Fig. 5H, Supplementary Table S5). The *Adgrg1*, *Pdcd1*, *Osgin1*, *Lag3*, and *Chn2* were predominantly expressed in tumor-reactive T cells. In particular, 89% of Adgrg1^+^CD8^+^ T cells were tumor-reactive T cells (Fig. 5I-K, Supplementary Table S6). Moreover, as all mice were under specific-pathogen-free (SPF) conditions without continuous virus antigen stimulation, the T cells with virus-specific TCR identified by trained prediction model (Supplementary methods) did not exhibit *Adgrg1* expression (Supplementary Figure S6, Supplementary methods).

In summary, the results obtained from Runx1^Runx1t1/+^; Mx1-Cre mouse validated the specific role of *ADGRG1* in tumor-reactive T cells of AML.

### ADGRG1^+^CD8^+^T cells were characterized as tumor-reactive T cells in AML with *RUNX1::RUNX1T1*

**Fig. 6:**
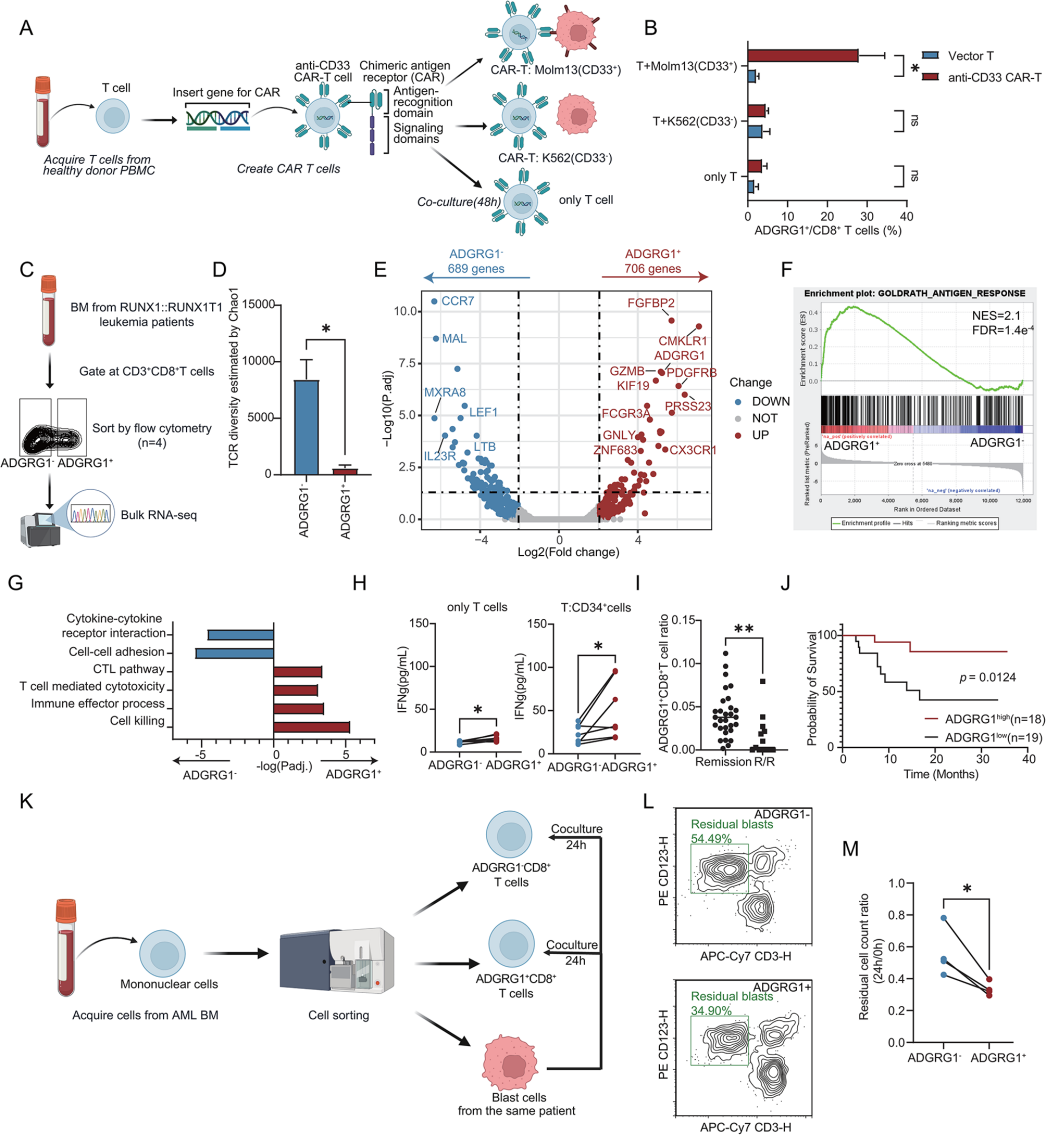
The function analysis of ADGRG1^+^CD8^+^T cells. **A** The coculture study workflow. T cells isolated from healthy donors’ peripheral blood were activated with CD3/CD28 Dynabeads and then transduced with anti-CD33 CAR-T lentivirus. On day 7 of production, anti-CD33 CAR-T cells were cocultured with AML cell line MOLM13 (CD33^+^) or K562 (CD33^−^) cells. **B** Bar plot showing the ADGRG1 expression detected by flow cytometry in CD8^+^ T cells. Blue represents vector T cells and red represents anti-CD33 CAR-T cells. **C** Schematic visualizing the sorting process for bulk RNA-seq from 4 AML BM. The ADGRG1^+^CD8^+^ T cells and ADGRG1^−^CD8^+^ T cells were sorted for following analysis. **D** The bar plot showing the TCR diversity of ADGRG1^−^ cells and ADGRG1^+^ cells reconstructed by TRUST4 [41]. **E** The volcano plot of the differentially expressed genes (DEGs) between ADGRG1^+^ cells (*n* = 3 after quality control) and ADGRG1^−^ cells (*n* = 4). The blue dots represent downregulated genes (689 genes), the red dots represent upregulated genes (708 genes), and the gray dots represent genes with no significant change. The vertical dashed line represents a log2 (fold change) value of 2, and the horizontal dashed line represents an adjusted p-value of 0.05. **F** Representative pathways enriched in the DEGs as determined by GSEA. The NES value and FDR value are annotated in the top right corner. **G** Bar plot showing the enriched pathways of DEGs. Blue represents pathways enriched in DEGs upregulated in ADGRG1^−^ cells and red represents pathways enriched in DEGs upregulated in ADGRG1^+^ cells. **H** The IFN-γ -releasing level of ADGRG1^+^CD8^+^ T cells and ADGRG1^−^CD8^+^ T cells from AML patients with *RUNX1::RUNX1T1*. The cells from the same patient are connected by lines. The IFN-γ was measured when T cells were cultured without blast cells on the left plot, and IFN-γ was measured 48 h after T cells cocultured with the corresponding patient’s BM CD34^+^ leukemia blast cells on the right plot. The p-value was calculated by paired t-test. **I** The ADGRG1^+^CD8^+^ T cell ratio calculated by deconvolution in remission patients and refractory/relapsed (R/R) patients so far. The p-value was performed by Student’s t-test. **J** Survival analysis of 37 AML patients with *RUNX1::RUNX1T1*. The p-value was determined by the log-rank test. **K** The cell killing assay workflow. Mononuclear cells were isolated from AML BM. ADGRG1^+^CD8^+^ T cells, ADGRG1^−^CD8^+^ T cells, and blast cells were sorted from each patient. Then ADGRG1^+/−^CD8^+^ T cells were cocultured with matched blasts with E: T = 1:1 for 24 h. **L** A representative flow cytometry plot of the cell killing assay result. The residual blasts are gated and the cell percentage is labeled in green. The X-axis represents CD3 (APC/Cy7) and the Y-axis represents CD123 (PE). **M** The residual blast cell count ratio between ADGRG1^−^ and ADGRG1^+^ groups. The cells from the same patient are connected by lines. The p-value was calculated by paired t-test. p-value: ns > 0.05, * ≤ 0.05, ** < 0.01, *** < 0.001, **** < 0.0001

Combining the results from both mouse and human samples mentioned above, we hypothesized that ADGRG1^+^CD8^+^ T cells in the BM of AML patients with *RUNX1::RUNX1T1* represent tumor-antigen-experienced cytotoxic T cells. To confirm this hypothesis, we first applied anti-CD33 CAR-T and Molm13 as an artificial effector-target cell model (Fig. 6A). When only under CD3/CD28 Dynabeads stimulation, neither CAR-T nor vector T cells showed significant ADGRG1 expression. After coculturing with K562 (CD33^−^), the anti-CD33 CAR-T also lacked ADGRG1 expression. However, under CD33 antigen stimulation (Molm13), the ADGRG1 was significantly upregulated on anti-CD33 CAR-T cells (*p* = 0.016, Fig. 6B, Supplementary Figure S7A), suggesting only the TCR-antigen interaction induces the evident ADGRG1 upregulation. For ADGRG1^+^CD8^+^ anti-CD33 CAR-T after coculture with Molm13, bulk RNA-seq data (Supplementary Figure S8, Supplementary Table S7) showed that its TCR signaling pathway was activated and the cytotoxic pathway was enriched, indicating higher cell-killing ability compared with ADGRG1^−^CD8^+^ anti-CD33 CAR-T cocultured with Molm13. Like the results in scRNA-seq, ADGRG1^+^CD8^+^ anti-CD33 CAR-T also showed NK-related features and were enriched in the senescence pathway.

To validate the findings in AML patients, we sorted ADGRG1^+/−^CD8^+^ T cells from 4 AML with *RUNX1::RUNX1T1* BM samples for bulk RNA-seq (Fig. 6C, Supplementary Figure S7B, Supplementary Table S8). After TCR reconstruction based on TRUST4 [41], we found that the TCR diversity of ADGRG1^+^CD8^+^ T cells was significantly lower than that of the ADGRG1^−^ group, indicating a state of TCR clonal expansion (Fig. 6D). Compared with the ADGRG1^−^ group, the ADGRG1^+^ group upregulated effector molecules like *GZMB*, NK-related genes like *FCGR3A*, Temra-related genes like *CX3CR1*, and *ADGRG1* upstream regulatory gene *ZNF683* [42] (Fig. 6E, Supplementary Table S9). Additionally, ADGRG1^+^CD8^+^ T cells exhibited antigen-response characteristics (Fig. 6F) and enriched pathways such as cytotoxicity and cell killing (Fig. 6G).

To prove that the upregulation of ADGRG1 in AML CD8^+^ T cells signifies tumor-reactive T cells, we isolated ADGRG1^+^CD8^+^ T cells and ADGRG1^−^CD8^+^ T cells from the BM samples of newly diagnosed AML patients with *RUNX1::RUNX1T1* (Supplementary Table S8) and co-cultured them with the matched patient’s BM CD34^+^ leukemia blast cells (Fig. 6H, Supplementary Figure S7C). We observed that ADGRG1^+^CD8^+^ T cells secreted higher levels of IFN-γ in the absence of leukemia cells. Upon co-culture 48 h with leukemia blasts, ADGRG1^+^CD8^+^ T cells exhibited increased IFN-γ secretion, significantly surpassing the levels observed in ADGRG1^−^CD8^+^ T cells, suggesting the tumor reactivity of ADGRG1^+^CD8^+^ T cells.

We also conducted a preliminary exploration into the clinical significance of the ADGRG1^+^CD8^+^ T cell subset. We collected BM samples from a total of 42 newly diagnosed *RUNX1::RUNX1T1* positive AML patients (Supplementary Table S10) administered in our center from September 2020 to October 2022 for bulk RNA-seq analysis. By deconvolution based on the single-cell landscape of AML patients (Supplementary Figure S9A-C, Supplementary methods, Supplementary information), the ADGRG1^+^CD8^+^ T cell ratios were calculated (Supplementary Fig. 9D). Relapsed/refractory patients had lower levels of ADGRG1^+^CD8^+^ T cells in their BM (*p* = 0.0048, Fig. 6I), suggesting that a lower proportion of these cells at the initial diagnosis might indicate a poor prognosis. Similarly, survival analysis showed that the ADGRG1^low^ group had a worse survival outcome (Fig. 6J).

### Characteristics of ADGRG1^+^CD8^+^T cells in myeloid neoplasms

To investigate whether ADGRG1^+^CD8^+^ T cells are specifically responsive to the *RUNX1::RUNX1T1* fusion protein, we used public scRNA-seq data for further analysis. The BM CD8^+^ T cell scRNA-seq data from AML/MDS patients (GSE250077) was integrated with scRNA-seq data from healthy donors [22]. In total, 23,654 CD8^+^ T cells were obtained (18,237 cells from AML/MDS patients and 5,417 cells from healthy donors). Dimension reduction, clustering, and cell annotation were performed as described workflow (Supplementary Figure S10A-C). The results showed that ADGRG1^+^CD8^+^ T cells were enriched in AML/MDS patients (Ro/e = 1.13) (Supplementary Figure S10D-E). *ADGRG1* was mainly expressed in Temra and Teff clusters (Supplementary Figure S10F). Similar to results from AML with *RUNX1::RUNX1T1*, ADGRG1^+^CD8^+^ T cells did not exhibit obvious elevated exhaustion scores but showed high cytotoxicity scores (Supplementary Figure S10G). In addition, ADGRG1^+^CD8^+^ T cells displayed upregulated cytotoxic molecules such as *GZMB* and NK-related markers like *KLRG1* (Supplementary Figure S10H). GSEA analysis showed that ADGRG1^+^CD8^+^ T cells enriched TCR signaling pathways and presented NK-like and senescence-like features (Supplementary Figure S10I). In addition, we analyzed the *Adgrg1* expression in the MLL-AF9 mouse model and found its significant upregulation in CD8^+^ Teff cells compared to that in the wild-type mice in public datasets [43] (Supplementary Figure S11).

Then we replicated the IFN-γ releasing assay in patients with other subtypes of AML to validate the function of ADGRG1^+^CD8^+^T cells (Supplementary Figure S7D, Supplementary Table S11). We obtained similar results compared with AML samples with *RUNX1::RUNX1T1*. Further, we sorted ADGRG1^+/−^CD8^+^ T cells from AML patients and cocultured them with matched blast cells for 24 h to evaluate their cell-killing ability (Fig. 6K, Supplementary Table S11). Results showed that the residual cells were significantly lower in the ADGRG1^+^ group (*p* = 0.0319), indicating its higher cell-killing ability (Fig. 6L-M). These suggested that the characteristic of *ADGRG1* as a marker for tumor-reactive T cells not only presented in *RUNX1::RUNX1T1* positive AML but also might be a common feature of tumor-reactive T cells in AML.

## Discussion

In this study, we characterized the heterogeneity of BM T cells in AML via paired scRNA-seq and scV(D)J-seq. In particular, we identified tumor-reactive T cells that exhibited distinctive non-exhausted profiles. Also, we recognized ADGRG1 as the specific marker of tumor-reactive T cells in AML. While additional experiments are necessary to confirm the genuine reactivity to AML blasts among TCR clones, the current definition methods are beneficial for our overall understanding of the traits of AML tumor-reactive T cells.

In solid tumors, tumor-reactive T cells are confined to a restricted microenvironment [44] and mainly exhibit terminally exhausted phenotypes [35]. However, our analysis shows the tumor-reactive T cells in AML lack classic exhaustion marker expression. Recently, a study has also suggested that the canonical exhausted T cells constituted less than 1% of all CD8^+^ T cells in AML [45]. Indeed, the depiction of T cell landscape in other hematologic malignancies has also revealed a limited presence of canonical exhausted T cells [32, 46]. Instead, the tumor-reactive T cells present as senescent-like cytotoxic Temra with upregulated NK markers, which has been reported as a novel dysregulation mechanism in CAR-T cells [47]. The killer cell lectin-like receptor-expressing cytotoxic phenotype has also been described as an alternate differentiation divergent from terminal CD8^+^ T cell exhaustion in relatively low antigen density niches [48]. This non-exhausted profile partially explains the reason that AML patients have little benefit from traditional immune checkpoint blockades (ICB) like anti-PD-1 therapy alone.

The identification of tumor-reactive TCR-engineered T cell (TCR-T) remains a time- and cost-consuming work [49]. To bypass the complexity of MHC typing and neoantigen prediction [50], we hope to identify characteristic markers of tumor-reactive T cells to enrich tumor-reactive TCRs. We find that ADGRG1 is specifically highly expressed in CD8^+^ tumor-reactive T cells compared to bystander T cells. Though AML with *RUNX1::RUNX1T1* patients were used as an example to explore, we also obtained consistent conclusions in other AML subtypes, suggesting that ADGRG1 could become a potential unified marker of CD8^+^ tumor-reactive T cells within AML. It could be further applied for tumor-reactive T cell enrichment, ex vivo expansion and adoptive cell therapy.

Besides serving as a marker for functional human hematopoietic stem cells [51] and leukemia stem cells [39], ADGRG1 also acts as a specific cell surface marker for cytotoxic lymphocytes [52]. In CD4^+^ T cells, researchers have found that KLRB1, KLRG1, and ADGRG1-positive T cells exhibit a high potential for tumor necrosis factor (TNF) / IFN-γ co-expression [53]. In cytotoxic NK cells, the expression of ADGRG1 is regulated by the transcriptional factor ZNF683 [42], which has also been upregulated in the tumor-reactive T cells from AML (Fig. 6E, Supplementary Table S2, S7, S9). ZNF683 regulates pathways of T cell activation/cytotoxicity and targets NK-like markers like KLRF1 through chromatin remodeling [54]. Upon antigen stimulation, it is possible that ADGRG1, as a member of the ZNF683 regulatory network, participates in the modulation of T cell effector function and NK transition, mediating the unique phenotype of AML tumor-reactive T cells. Therefore, the selective expression of ADGRG1 on CD8^+^ T cells can function as an effective indicator of TCR signal activation [55]. While previous studies have reported elevated baseline levels of ADGRG1 in T cells from cytomegalovirus-infected (CMV) patients [40, 52], our data demonstrate that T cells solely possessing virus-specific TCR do not express ADGRG1 (Supplementary Figure S6), indicating that the prerequisite for ADGRG1 expression is continuous antigen experience. For AML patients with latent CMV infection, as the CMV TCR repertoire has been studied [56], it is feasible to exclude them when screening tumor-reactive TCRs.

Meanwhile, we found that relapsed/refractory AML patients have fewer ADGRG1^+^CD8^+^ T cells at diagnosis, suggesting the lack of a robust anti-tumor immune response. Therefore, the proportion of ADGRG1^+^CD8^+^ T cells at diagnosis may serve as an early biomarker for prognosis prediction. Given the small cohort size and limited observation period, this observation needs to be followed up with larger prospective cohorts. Moreover, a larger scale of analysis across different AML subtypes is needed to confirm the role of ADGRG1 in CD8^+^ tumor-reactive T cells.

Presently, the development of T-cell therapy for AML is still underway with insufficient effectiveness. By drawing on experiences from solid tumor research, we identified tumor-reactive T cells with unique phenotypes in AML, especially AML with *RUNX1::RUNX1T1*, and detected ADGRG1 as their marker. Our findings contribute to a better understanding of the immune editing mechanisms in AML and provide a simpler, more feasible approach for future tumor-reactive TCR screening.

## Electronic supplementary material

Below is the link to the electronic supplementary material.

Supplementary Material 1

Supplementary Material 2

Supplementary Material 3

Supplementary Material 4

Supplementary Material 5

Supplementary Material 6

Supplementary Material 7

Supplementary Material 8

Supplementary Material 9

Supplementary Material 10

Supplementary Material 11

Supplementary Material 12

Supplementary Material 13

## Data Availability

The human raw sequence data reported in this paper have been deposited in the Genome Sequence Archive in National Genomics Data Center, China National Center for Bioinformation/Beijing Institute of Genomics, Chinese Academy of Sciences (GSA-Human: HRA005884, HRA007077) that are publicly accessible at https://www.ngdc.cncb.ac.cn/gsa-human. The mouse data discussed in this publication have been deposited in NCBI’s Gene Expression Omnibus and are accessible through GEO Series accession number GSE263099 (https://www.ncbi.nlm.nih.gov/geo/query/acc.cgi?acc=GSE263099). The human healthy donors’ BM data are obtained from GSE120221. The AML/MDS data are obtained from GSE250077. The MLL-AF9 mouse data are obtained from GSE240025 [43]. Other detailed data supporting this study’s findings are available from the corresponding author upon reasonable request.
