# Supplementary material for: Identifying ADGRG1 as a specific marker for tumor-reactive T cells in acute myeloid leukemia
Source: Exp Hematol Oncol. 2024 Sep 6;13:92. doi: 10.1186/s40164-024-00560-0 (PMC11380426; doi:10.1186/s40164-024-00560-0)
Supplement: Supplementary file 2 — Supplementary Material 2 [file 40164_2024_560_MOESM2_ESM.docx]

Supplementary methods

Generation of Runx1^Runx1t1/+^; Mx1-Cre mouse model

The Runx1^Runx1t1/+^; Mx1-Cre were generated by breeding Runx1^Runx1t1/+^ mice with Mx1-Cre transgenic mice by Shanghai Model Organisms Center, Inc (Shanghai, China). The Runx1^Runx1t1/+^; w/o Mx1-Cre (control group) and Runx1^Runx1t1/+^; Mx1-Cre (Runx1::Runx1t1 group) mice used in each experiment were sex-matched littermates. Mice were given 5 mg/kg body weight of GE Polyinosine-polycytosine (Poly[I:C], Cat# 27473201) every other day for consecutive 4 times by intraperitoneal (i.p.) injection to induce Cre expression. The mice were sacrificed for the following experiments four weeks after Poly[I:C] induction. The absolute number of long-term hematopoietic stem cells (LT-HSCs), short-term hematopoietic stem cells (ST-HSCs), and multipotent progenitors (MPPs) in the bone marrow (BM) increased significantly in the RUNX1::RUNX1T1 group compared with that of the control group, while common myeloid progenitors (CMPs), granulocyte/monocyte progenitors (GMPs), megakaryocyte/erythroid progenitor s(MEPs), myeloid and erythroid cells decreased obviously, presenting a significant excessive accumulation of aberrant HSCs and the differentiation blockage profile. Two mouse BM were mixed as one biological duplicate and each group in scRNA-seq included two biological duplicates. All mouse-related experimental procedures underwent review and approval by the ethics committee.

Preparation of cell suspensions from human bone marrow for scRNA-seq

The density gradient centrifugation isolated bone marrow mononuclear cryopreserved samples were thawed and washed twice with RPMI-1640 containing 10% fetal bovine serum (FBS) and filtered through a 40 µm strainer before flow cytometry sort. Cells were stained with the antibody against human CD45 (Biolegend, Cat# 982316), CD3 (Biolegend, Cat# 317317), CD4 (Biolegend, Cat# 300511), CD8 (Biolegend, Cat# 300921), and DAPI (Sigma-Aldrich, Cat# D9542). DAPI^-^CD45^high^SSC^low^CD3^+^CD4^+^CD8^-^ cells and DAPI^-^CD45^high^SSC^low^CD3^+^CD4^-^CD8^+^ cells were sorted and mixed as T cells for sequencing.

Preparation of cell suspensions from mouse bone marrow for scRNA-seq

To harvest bone marrow, place the euthanized mouse in a supine position and sterilize the skin using ethanol. The ilium\femur\fibula bones were cut off, ground in cold complete buffer (PBS with 2% FBS and 10mmol/L EDTA), and filtered through a 40 µm strainer. Then, gradient centrifugation (400G, 5min) was carried out and resuspended. EasySep™ Mouse T Cell Isolation Kit (STEMCELL, Cat # 19851) was applied to T cell negative enrichment. The enriched T cells were stained with the antibody against mouse CD3 (Biolegend, Cat# 100235) and DAPI. DAPI^-^ mCherry^-^ CD3^+^ cells were sorted in both groups by flow cytometry with a BD FACSAria™ III sorter (BD Biosciences) for the scRNA-seq library.

Processing and quality control of single-cell RNA-seq data.

For single-cell RNA-seq data, human raw reads obtained from the 10× Genomics were demultiplexed and mapped to the human reference genome GRCh38 using the Cell Ranger toolkit (version 6.1.2). Similarly, mouse raw reads were demultiplexed and mapped to the mouse reference genome GRCm38. Then cells with fewer than 200 genes detected or > 15% mitochondrial UMI counts were filtered out by Scanpy (version 1.9.1)[1]. Scrublet, the wrapper function from Scanpy, was applied to each sequencing library to remove potential doublets, setting the expected doublet rate to 0.05, and the predicted doublets were filtered out. The normalized expression matrix was calculated based on the raw UMI counts after normalizing total counts per cell (library size) and then scaled by 1e6 and logarithmically transformed by *scanpy.pp.normalize_total()* and *scanpy.pp.log1p()* function.

Dimension reduction and unsupervised clustering for scRNA-seq data

After quality control, the *scanpy.pp.highly_variable_genes()* function was performed to select the top 4,000 highly-variable genes (HVGs). Then, the effects of the total counts per cell and the percentage of mitochondrial gene counts were regressed out by the *scanpy.pp.regress_out()* function. The *scanpy.tl.pca()* function with parameter *svd_solver=’arpack’* was then applied to principal component analysis (PCA) which calculates the main axes of variation and denoises the data. To correct the batch effects from different samples, we applied BBKNN[2] to generate a batch-balanced k nearest neighbor (KNN) graph, which identifies the top neighbors of each cell in each batch separately. The *scanpy.tl.umap()* function was used for non-linear dimension reduction to generate the Uniform Manifold Approximation and Projection (UMAP) visualization. To cluster single cells by their expression profiles, we used an unsupervised graph-based clustering algorithm, Leiden, with the *scanpy.tl.leiden()* function.

After the first round of unsupervised clustering, the CD3^+^CD8^+^CD4^-^ and CD3^+^CD8^-^CD4^+^ T cells were isolated based on their average gene expression (*CD3D*, *CD3G*, *CD3E*, *CD8A*, *CD8B*, *CD4*) and were processed separately in downstream clustering and analysis.

To reduce noise and to obtain the high-resolution map, we performed two-run clustering on each dataset. The immunoglobulin genes, T cell receptor (TCR) genes, ribosome-protein-coding genes, and proliferation genes were excluded when performing clustering[3]. The second-round clustering procedure was the same as the first-round clustering, including HVGs identification, PCA matrix calculation, batch effects calculation by BBKNN, cell cluster detection by the Leiden algorithm, and dimension reduction for visualization. Marker genes and differentially expressed genes (DEGs) were detected using the *scanpy.tl.rank_genes_groups()* function with the instructed parameter.

TCR sequences assembly

For scV(D)J-seq data, the Cell Ranger toolkit (version 6.1.2) applied TCR-seq reads to the human reference genome (GRCh38) or mouse reference genome (GRCm38) to assemble TCR sequences. A Python-based toolkit, Scirpy (version 0.11.0)[4], was used for the TCR filter. The preliminary TCR sequences were filtered to keep those characterized with high confidence, full-length, productive, and assigned with a valid cell barcode and an unambiguous chain type. The *scirpy.pp.merge_with_ir()* function was used to merge TCR information with scRNA-seq data. The function *scirpy.tl.define_clonotypes()* matches cells based on the distances of their VJ and VDJ CDR3 base sequences and the value of the function parameters *dual_ir=’primary_only’* and *receptor_arms=’all’*. Each cell was assigned one pair of alpha and beta chains with the highest UMI counts. The multichain (> 2) and the orphan chain were filtered. Cells with identical TCR pairs were defined as clonotype and considered to originate from the same ancestry.

For clonotype found in both CD4^+^ and CD8^+^ T cells, the ratio of cell numbers between CD4^+^ and CD8^+^ T cells was calculated and the exclusion criteria are detailed in the previous study[3].

Single-cell trajectory analysis and definition of cell states

The interested biologically relevant subgroups, such as CD8^+^ T cells, were selected for single-cell trajectory analysis. By the function *scanpy.pp.neighbors()* and *sc.tl.diffmap()*, the cell groups were reduced dimension into diffusion map. Then the pseudo-time was calculated by Palantir (version 1.3.2)[5] with *palantir.core.run_palantir()* function. The root cell was artificially designated with biological significance, such as naïve T, and the terminal cell state was automatically calculated.

The identification of potentially tumor-reactive T cells (pTRTs)

To distinguish the T cells reacting to tumors from bystander T cells, we referenced the approach of identifying tumor-reactive T cells in STARTRAC[6] and applied it to our Python analysis workflow for AML. Mainly, we jointly analyzed the features of clonal expansion, proliferation, tumor enrichment, and TCR downstream signal activation. In the mouse model, the pTRT identification process is similar to that in human samples.

1. The clonal expansion index

The Shannon entropy is a measure of the uncertainty or randomness in a system and can be used to measure the distribution evenness of the TCR repertoire. The normalized Shannon entropy was calculated by the *scirpy.tl.alpha_diversity()* function which normalized the Shannon entropy to group size. The clonal expansion index was as “1- normalized Shannon entropy” and the high value indicated high clonality of each cluster.

1. The proliferation index

The proliferation index was used to complement the TCR-based clonal expansion index and indicated the ongoing proliferation activity of a cluster. For each cell, a proliferating score was calculated by the *scanpy.tl.score_genes()* function, which was the average expression of a set of proliferation markers (*ZWINT*, *E2F1*, *FEN1*, *FOXM1*, *H2AFZ*, *HMGB2*, *MCM2*, *MCM3*, *MCM4*, *MCM5*, *MCM6*, *MKI67*, *MYBL2*, *PCNA*, *PLK1*, *CCND1*, *AURKA*, *BUB1*, *TOP2A*, *TYMS*, *DEK*, *CCNB1*, *CCNE1*) subtracted with the average expression of a reference set of randomly sampled genes. As the distribution of the proliferating score approximated a normal distribution, the proliferating cells were defined as those with a $z score=\frac{Observation-Mean}{Standard deviation}$ of proliferating score falling outside of 1.645 standard deviation. The proliferation index was calculated as the frequency of proliferating cells in each cluster.

1. The tumor enrichment index

To quantify the enrichment of immune cell clusters between the tumor and normal group, the observed and expected frequency for each cluster in each group as previously described[7].

$$Tumor enrichment index (Ro/e)=\frac{Observed frequency in tumor group}{Expected frequency in tumor group}$$

The expected frequency was obtained from the Chi-square test via the *chi2_contingency()* function from Scipy (version 1.7.3). If the tumor-enrichment index > 1, we assumed that the cluster was more enriched in the tumor group.

1. The TCR downstream signal activation

The antigen-reactive T cells tend to exhibit high activities in TCR signaling. To value the TCR signaling pathway, we select TCR-signaling-related genesets from MsigDB (<https://www.gsea-msigdb.org/gsea/msigdb>). The AUC score of each cell in every gene set was obtained by AUCell (<https://github.com/aertslab/AUCell>, version 1.12.0). The distribution of AUC scores across all the cells allows for exploring the relative activity of the TCR signaling pathway.

**The exploration of maker genes in tumor-reactive T cells**

To explore the distinctive maker of tumor-reactive T cells in AML, we first examined the average gene expression pattern of tumor-reactive T cells and bystander T cells. For the top 100 genes based on the delta value (delta = average expression in tumor-reactive T cells - average expression in bystander T cells), the proportion of tumor-reactive T cells among the cells expressing those genes was calculated and sorted by descending to evaluate the specificity.

**The protein-protein interaction network analysis**

The genes upregulated in the ADGRG1^+^ group were obtained and inputted into the STRING database[8] to get the protein-protein interactions (PPI) with a 0.4 (medium confidence) minimum required interaction score. The Cytoscape software (version 3.9.1)[9] was used for the following interaction network visualization.

The prediction of virus-specific TCR clonotypes

Regarding the Runx1^Runx1t1/+^; Mx1-Cre mouse was C57BL/6 background with H2-K^b^ and H2-D^b^ as their MHC Class I molecules, the training CDR3 dataset was obtained from VDJdb (<https://vdjdb.cdr3.net/search>) and filtered by 1) confidence score > 0; 2) epitopes that have at least 50 paired α and β chains; 3)antigen presented by H2-K^b^ and H2-D^b^. Finally, 6 epitope prediction models(m139, M38, NP, PA, M45, PB1) were constructed based on the CDR3 sequence of paired α and β chains by TCRGP (version 1.0.0)[10] with parameters *iteration=5000* and *learning rate=0.005*. Then epitope predictions were performed on the single cell V(D)J data. The probability needed to be above a cut-off of 0.9 to be considered as specific to the tested epitope and 4 epitopes (PA, PB1, M45, m139) were recognized.

Bulk RNA-seq and data analysis

The total RNA was extracted from samples using TriZol Reagent (TaKaRa, Cat# 9109). For the data sequenced in clinical, we employed a ribosomal RNA depletion method for library preparation and sequenced in the Illumina NovaSeq 6000 platform. The adapters were removed from Fastq data by Trimmomatic (Version 0.39)[11]. Then the clean data was qualified by FastQC software (Version 0.11.9, <https://www.bioinformatics.babraham.ac.uk/projects/fastqc/> ) and mapped to the UCSC human reference genome (hg19) using STAR (version 2.7.8a)[12]. The mapped reads for genomic features were counted by Subread (version 2.0.6)[13] via the *featureCounts()* function. The steps of data processed and sequenced in the laboratory were almost the same as clinical sequenced data, except the library was prepared using the PolyA enrichment method. Then count matrix was counted by the Python package rnanorm (version 2.1.0, <https://github.com/genialis/RNAnorm>) with the parameter *--fpkm-output* to generate the fragments per kilobase of exon model per million mapped fragments (FPKM) matrix. The differentially expressed genes were calculated by DEseq2 (version 3.18)[14], enriched by GSEA (version 4.3.2)[15], and the volcano plot was performed by ggplot2 (version 3.5.0, <https://ggplot2.tidyverse.org/> ). To reconstruct TCR information from RNA-seq data, the BAM files were analyzed by TRUST4 (version 1.0.7)[16].

**The deconvolution analysis of AML bulk RNA-seq data**

We integrate healthy donor bone marrow and our AML bone marrow scRNA-seq as a reference (**Supplementary Figure S9**, **Supplementary information**). The analysis workflow was performed by BayesPrism (version 1.3)[17] following their instructions. After cell type quality control and outlier gene filtering, the Prism object was created by *new.prism()* function with default parameters. The prism object contained the scRNA-seq reference matrix, the cell type labels of each row of reference, and the mixture matrix for bulk. Then, 42 newly diagnosed AML bone marrow with *RUNX1::RUNX1T1* bulk RNA-seq count matrices were deconvoluted through *run.prism()* function. The proportion of ADGRG1^+^CD8^+^ T cell group was estimated between remission and relapsed/refractory groups. Among them, 37 patients had survival information. We ranked these patients based on the deconvoluted proportion of ADGRG1^+^CD8^+^ T cells, categorizing them into the ADGRG1^high^ and the ADGRG1^low^ groups (**Supplementary Figure S9D**). Then survival curves were plotted using disease relapse as the endpoint and statistical analysis was performed by the log-rank test.

Supplementary information

Generation of the single-cell reference map for deconvolution

To construct a single-cell reference map for deconvolution, we collected a total of 113,089 bone marrow cells, including 74,865 total bone marrow cells from healthy donors[18], 11,311 CD34^+^ blast cells from AML patients with *RUNX1::RUNX1T1*, and 26,913 T cells from AML patients with *RUNX1::RUNX1T1*.

Through unsupervised clustering analysis, a total of 16 subgroups were identified with specific markers (**Supplementary Figure S9B-C**). The *CD34^+^* cells from AML patients were identified as the blast group, while *CD38^+^* cells from healthy donors were annotated as hematopoietic progenitor cells (HPC). Those *CD3E^+^* cells were characterized as T cells including naïve T (Tn, *CCR7*), central memory T cells (Tcm, *CD44*), mucosal-associated invariant T cells (MAIT, *SLC4A10*), effector T cells (Teff, *IFNG*), and ADGRG1^+^CD8^+^T cells (*CX3CR1*, *ADGRG1*). Cells with *CD19* and *MS4A1* expression were identified as B cells, cells with *CD28* were identified as plasma cells, while cells with *IGLL1* expression were B progenitor cells. The Ery group was characterized by erythroid markers like *HBD*. In addition, there were myeloid cells with specific markers, including monocytes (*LYZ*) and dendritic cells (DC, *FCER1A*). The 16 clusters were used as reference data for deconvolution.

Supplementary figure legends

**Supplementary Fig. 1 The transcriptional features of CD8^+^ and CD4^+^ T cells in the BM of AML with *RUNX1::RUNX1T1*. A** UMAP visualization of BM CD8^+^ T cells. Orange represents the RUNX1::RUNX1T1 group and green represents the HD group. **B** The feature plots showing the expression levels of selected genes. **C** The ribbon plot showing the V(D)J gene usage of the MAIT cluster. **D** The diffusion map of CD8^+^ T cells. The clusters corresponding to each color in the UMAP plot are indicated in the right-side legend. **E** The diffusion map of the HD group (left) and the RUNX1::RUNX1T1 group (right). The color shows the pseudotime performed by Palantir and the color bar is exhibited on the right margin. **F** UMAP visualization of identified 5 CD4^+^ T cell clusters. The subgroups corresponding to each color in the UMAP plot are indicated in the right-side legend. **G** UMAP visualization of BM CD4^+^ T cells. Yellow represents the RUNX1::RUNX1T1 group and green represents the HD group. **H** Dot plot showing the relative expression of marker genes across different CD4^+^ T cells. Bubble size is proportional to the percentage of cells expressing a gene and color intensity is proportional to average scaled gene expression. **I** Boxplot showing the proportion of CD4^+^ T cell in RUNX1::RUNX1T1 (red) and HD (blue) group. Student’s t-test was used to measure the differences between the two groups.

**Supplementary Fig. 2 The index used for pTRT evaluation of CD8^+^ T cells. A** The bar plot showing the tumor enrichment index in CD8^+^ T cell clusters. The high-score clusters are shown in red. **B** The bar plot showing the clonal expansion index in CD8^+^ T cell clusters. The high-score clusters are shown in red. **C** The bar plot showing the proliferation index in CD8^+^ T cell clusters. The high-score clusters are shown in red. **D** The violin plot of NeoTCR score in CD8^+^ T cell clusters. The NeoTCR score is calculated by gene sets published by Frank J Lowery et al[19]. **E** The UMAP plot showing the original clusters. Due to the distinct lineage origin of MAIT cells as evidenced by their TCR sequences, the MAIT cells were not incorporated in the dimension reduction and re-clustering process. **F** The box plot showing the fractions of original clusters within these newly defined clusters. **G** The UMAP plot illustrating the pseudotime performed by Palantir and the color bar is exhibited on the right margin.

**Supplementary Fig. 3 The ribbon plot showing the V(D)J gene usage of the tumor-reactive T cells.** The most frequently used gene is labeled on the plot.

**Supplementary Fig. 4 The ADGRG1/Adgrg1 expression profile in healthy people or wild-type mice A** The bar plot showing the human ADGRG1 expression profile in GSE120221[18]. **B** The bar plot showing the human ADGRG1 expression profile in the human cell atlas database[20]. **C** The bar plot showing the mice's Adgrg1 expression profile in the GSE122465[21]. **D** The bar plot showing the mice’s Adgrg1 expression profile in the GSE124822-1[22]. The y-axis represents the expression level of ADGRG1, while the x-axis represents different cell clusters (based on the cell annotation in the original article).

**Supplementary Fig. 5 The transcriptional features of Cd8^+^ and Cd4^+^ in the Runx1::Runx1t1 mouse model.** **A** Dot plot showing the relative expression of marker genes across different Cd8^+^ T cells. **B** Boxplot showing the proportion of Cd8^+^ T cell in the Runx1::Runx1t1 group (red) and the control (blue) group. **C** Violin plot showing the senescence score among pTRT-irrelevant, pTRT-relevant, and pTRT. The Wilcoxon rank-sum test was applied to calculate the p-value between pTRT/pTRT-relevant group with pTRT-irrelevant group (**** ≤ 0.0001). **D** UMAP visualization of identified 15 Cd4+ T cell clusters. **E** Dot plot showing the relative expression of marker genes across different Cd4^+^ T cells. **F** The ribbon plot showing the V(D)J gene usage of the iNKT cluster. **G** Boxplot showing the proportion of Cd4^+^ T cell in Runx1::Runx1t1 group (red) and control (blue) group.

**Supplementary Fig. 6 The identification of virus-specific T cells in the Runx1::Runx1t1 mouse models.** **A** ROC curves of four virus epitope recognition prediction models. These models were built using data from validated and paired TCR ɑ and β chain which recognized virus antigen peptides presented by MHC molecules unique to C57BL/6 background mice (H-2Kb and H-2Db) as a training set. The virus epitope is annotated on the top of each plot. **B** UMAP visualization of identified virus-specific T cells. The recognized epitope and corresponding virus source are shown on the right margin. **C** The violin plot showing the *Adgrg1* expression level among the tumor-reactive T cells, virus-specific T cells, and bystander cells. **D** The matrix plot showing the distribution prevalence of Adgrg1^+/-^Cd8^+^ T cells estimated by Ro/e among the tumor-reactive T cells, virus-specific T cells, and bystander cells. The color bar is shown on the right side of the plot.

**Supplementary Fig. 7 The characteristics of ADGRG1^+^ CD8^+^ T cells.** **A** An example of the flow cytometry plot of the coculture result. The ADGRG1^+^ fraction is gated as R5. **B** The fragments per kilobase per million (FPKM) value of ADGRG1^-^ group (n=4) and ADGRG^+^ group (n=3 after quality control) which validated the flow cytometry gating strategy. **C** The ridge plot showing the IFN-γ fluorescence intensity in different culture groups. **D** The IFN-γ -releasing level of ADGRG1^+^CD8^+^ T cells and ADGRG1^-^CD8^+^ T cells from AML patients. The cells from the same patient are connected by lines. IFN-γ was measured when T cells were cultured without blast cells on the left plot (Left). IFN-γ was measured 48h after T cells cocultured with the corresponding patient's BM CD34^+^ leukemia blast cells on the right plot (Right). The p-value was calculated by paired t-test. p value: * ≤ 0.05.

**Supplementary Fig. 8 The RNA characteristics of ADGRG1^+/-^CD8^+^ anti-CD33 CAR-T cells. A** PCA plot showing the cluster of bulk RNA-seq samples. T cells from 4 different healthy donors were used to construct anti-CD33 CAR-T and then cocultured with Molm13. After 24h, ADGRG1^+/-^CD8^+^ anti-CD33 CAR-T were sorted respectively for bulk RNA-seq. PCA analysis showed that ADGRG1^-^ and ADGRG1^+^ CAR-T cells were clustered separately. **B** The count value of the ADGRG1^+^CD8^+^ anti-CD33 CAR-T group and ADGRG1^-^CD8^+^ anti-CD33 CAR-T group which validated the flow cytometry gating strategy. **C** The volcano plot of the differentially expressed genes (DEGs) between the ADGRG1^+^CD8^+^ anti-CD33 CAR-T group and ADGRG1^-^CD8^+^ anti-CD33 CAR-T group. The blue dots represent downregulated genes (165 genes), the red dots represent upregulated genes (228 genes), and the gray dots represent genes with no significant change. The vertical dashed line represents a log2 (fold change) value of 1, and the horizontal dashed line represents an adjusted p-value of 0.05. **D-H** Representative pathways enriched in the DEGs as determined by GSEA. The NES value and FDR value are annotated in the top right corner.

**Supplementary Fig. 9 The reference single-cell transcriptome profile for deconvolution.** **A** Schema of deconvolution analysis. The bulk RNA-seq data was inputted and deconvoluted based on scRNA-seq data. Then the cluster percentages were inferred. Patients were categorized into different groups for the following analysis like survival analysis. **B** Dot plot showing the relative expression of marker genes for cell annotation. Bubble size is proportional to the percentage of cells expressing a gene and color intensity is proportional to average scaled gene expression. **C** UMAP showing the identified 16 clusters in BM. The subgroups corresponding to each color in the UMAP plot are indicated in the right-side legend. **D** The bar plot showing the ADGRG1^+^CD8^+^ T cell ratio calculated by deconvolution. The ADGRG1^high^ groups (red) and the ADGRG1^low^ groups (white) were separated by the medium value of ADGRG1^+^CD8^+^ T cell ratio.

**Supplementary Fig. 10 The *ADGRG1* expression pattern in AML/MDS BM CD8^+^ T cells from public data. A** UMAP visualization of identified 9 CD8^+^ T cell clusters. The scRNA-seq data were obtained from GSE250077 and GSE120221. The subgroups corresponding to each color in the UMAP plot are indicated in the right-side legend. **B** Dot plot showing the relative expression of marker genes across different CD8^+^ T cells. Bubble size is proportional to the percentage of cells expressing a gene and color intensity is proportional to average scaled gene expression. **C** UMAP plot showing the cell group. The dark blue represents AML/MDS group and light blue represents the HD group. **D** The UMAP plot visualizing the *ADGRG1* expression in CD8^+^ T cells. **E** The distribution pattern of ADGRG1^+/-^CD8^+^ T cells. Matrix plot showing distribution prevalence estimated by Ro/e. **F** Dot plot showing the relative expression of specific markers across different clusters. **G** The UMAP plot illustrating the cytotoxicity and exhaustion score in CD8^+^ T cells. The color bar is shown on the right margin. **H** The volcano plot of the differentially expressed genes (DEGs) between ADGRG1^+^ cells and ADGRG1^-^ cells. The blue dots represent downregulated genes (60 genes), the red dots represent upregulated genes (1154 genes), and the gray dots represent genes with no significant change. The vertical dashed line represents a log2 (fold change) value of 1, and the horizontal dashed line represents an adjusted p-value of 0.05. **I** Representative pathways enriched in the DEGs as determined by GSEA. The NES value and FDR value are annotated in the top right corner.

**Supplementary Fig. 11 The Adgrg1 expression level in the MLL-AF9 mouse model.** The data were obtained from public data[23]. WT: wild type mice. The p-value was calculated by t-test. p-value: ** < 0.01.

**References**

1. Wolf FA, Angerer P, Theis FJ. SCANPY: large-scale single-cell gene expression data analysis. Genome Biol. 2018;19:15.

2. Polański K, Young MD, Miao Z, Meyer KB, Teichmann SA, Park J-E. BBKNN: fast batch alignment of single cell transcriptomes. Bioinforma Oxf Engl. 2020;36:964–5.

3. Zheng L, Qin S, Si W, Wang A, Xing B, Gao R, et al. Pan-cancer single-cell landscape of tumor-infiltrating T cells. Science. 2021;374:abe6474.

4. Sturm G, Szabo T, Fotakis G, Haider M, Rieder D, Trajanoski Z, et al. Scirpy: a Scanpy extension for analyzing single-cell T-cell receptor-sequencing data. Bioinforma Oxf Engl. 2020;36:4817–8.

5. Setty M, Kiseliovas V, Levine J, Gayoso A, Mazutis L, Pe’er D. Characterization of cell fate probabilities in single-cell data with Palantir. Nat Biotechnol. 2019;37:451–60.

6. Zhang L, Yu X, Zheng L, Zhang Y, Li Y, Fang Q, et al. Lineage tracking reveals dynamic relationships of T cells in colorectal cancer. Nature. 2018;564:268–72.

7. Liu Y, Zhang Q, Xing B, Luo N, Gao R, Yu K, et al. Immune phenotypic linkage between colorectal cancer and liver metastasis. Cancer Cell. 2022;40:424-437.e5.

8. Szklarczyk D, Kirsch R, Koutrouli M, Nastou K, Mehryary F, Hachilif R, et al. The STRING database in 2023: protein-protein association networks and functional enrichment analyses for any sequenced genome of interest. Nucleic Acids Res. 2023;51:D638–46.

9. Shannon P, Markiel A, Ozier O, Baliga NS, Wang JT, Ramage D, et al. Cytoscape: A Software Environment for Integrated Models of Biomolecular Interaction Networks. Genome Res. 2003;13:2498–504.

10. Jokinen E, Huuhtanen J, Mustjoki S, Heinonen M, Lähdesmäki H. Predicting recognition between T cell receptors and epitopes with TCRGP. PLoS Comput Biol. 2021;17:e1008814.

11. Bolger AM, Lohse M, Usadel B. Trimmomatic: a flexible trimmer for Illumina sequence data. Bioinformatics. 2014;30:2114–20.

12. Dobin A, Davis CA, Schlesinger F, Drenkow J, Zaleski C, Jha S, et al. STAR: ultrafast universal RNA-seq aligner. Bioinforma Oxf Engl. 2013;29:15–21.

13. Liao Y, Smyth GK, Shi W. The R package Rsubread is easier, faster, cheaper and better for alignment and quantification of RNA sequencing reads. Nucleic Acids Res. 2019;47:e47.

14. Love MI, Huber W, Anders S. Moderated estimation of fold change and dispersion for RNA-seq data with DESeq2. Genome Biol. 2014;15:550.

15. Subramanian A, Tamayo P, Mootha VK, Mukherjee S, Ebert BL, Gillette MA, et al. Gene set enrichment analysis: a knowledge-based approach for interpreting genome-wide expression profiles. Proc Natl Acad Sci U S A. 2005;102:15545–50.

16. Song L, Cohen D, Ouyang Z, Cao Y, Hu X, Liu XS. TRUST4: immune repertoire reconstruction from bulk and single-cell RNA-seq data. Nat Methods. 2021;18:627–30.

17. Chu T, Wang Z, Pe’er D, Danko CG. Cell type and gene expression deconvolution with BayesPrism enables Bayesian integrative analysis across bulk and single-cell RNA sequencing in oncology. Nat Cancer. 2022;3:505–17.

18. Oetjen KA, Lindblad KE, Goswami M, Gui G, Dagur PK, Lai C, et al. Human bone marrow assessment by single-cell RNA sequencing, mass cytometry, and flow cytometry. JCI Insight. 2018;3:e124928, 124928.

19. Lowery FJ, Krishna S, Yossef R, Parikh NB, Chatani PD, Zacharakis N, et al. Molecular signatures of antitumor neoantigen-reactive T cells from metastatic human cancers. Science. 2022;375:877–84.

20. Regev A, Teichmann SA, Lander ES, Amit I, Benoist C, Birney E, et al. The Human Cell Atlas. eLife. 2017;6:e27041.

21. Kurtova AV, Heinlein M, Haas S, Velten L, Dijkgraaf GJP, Storm EE, et al. Disruption of stem cell niche-confined R-spondin 3 expression leads to impaired hematopoiesis. Blood Adv. 2023;7:491–507.

22. Izzo F, Lee SC, Poran A, Chaligne R, Gaiti F, Gross B, et al. DNA methylation disruption reshapes the hematopoietic differentiation landscape. Nat Genet. 2020;52:378–87.

23. Shi R-Y, Zhou N, Xuan L, Jiang Z-H, Xia J, Zhu J-M, et al. Trafficking circuit of CD8+ T cells between the intestine and bone marrow governs antitumour immunity. Nat Cell Biol. 2024;
